# Patterns of ambulatory care utilization in Taiwan

**DOI:** 10.1186/1472-6963-6-54

**Published:** 2006-05-04

**Authors:** Tzeng-Ji Chen, Li-Fang Chou, Shinn-Jang Hwang

**Affiliations:** 1 Department of Family Medicine, Taipei Veterans General Hospital, Shih-Pai Road, Section 2, No 201, Taipei 11217, Taiwan; 2 National Yang-Ming University School of Medicine, Taipei, Taiwan; 3 Department of Public Finance, National Chengchi University, Taipei, Taiwan

## Abstract

**Background:**

We used the insurance claims of a representative cohort to quantify the patterns of ambulatory care visits, especially the doctor-shopping phenomenon, in Taiwan.

**Methods:**

The ambulatory visit files of the 200,000-person cohort datasets from the National Health Insurance Research Database in 2002 were analyzed. Only a visit with physician consultation would be considered. We computed the visit patterns both by visit count and by patient count.

**Results:**

In 2002, there were 182,474 eligible people with 2,443,003 physician consultations. During the year, 87.4% of the cohort had visited physician clinics and 57.5% had visited hospital-based outpatient or emergency departments. On average, a person had 13.4 physician consultations and consulted 3.4 specialties, 5.2 physicians, and 3.9 healthcare facilities in a year. In 2002, 17.3% of the cohort had ever visited different healthcare facilities on the same day; 23.5% had ever visited physicians of the same specialty at different healthcare facilities within 7 days and the percentage of second visits was 3.8% of all visits. Besides, 7.6% of the cohort had visited two or more specialties at the same facility on the same day, and such visits make up 2.5% of all visits.

**Conclusion:**

The people in Taiwan did visit the physicians and outpatient departments frequently. Many patients not only consulted several physicians of different specialties and at different healthcare facilities during the year, but also switched the physicians and facilities quickly. An effective referral system with efficient data exchange between facilities might be the solution.

## Background

The people in Taiwan are occasionally criticized for having the habit of "doctor-shopping", implicating frequent attendances and switching of physicians. This phenomenon has been also reported in Japan [1] and Hong Kong [2,3]. No concrete, large-scale data are yet available to describe the patterns of physician service utilization in Taiwan. Although the health authorities in Taiwan release statistical reports periodically, the official statistics offer only aggregate data from the administrative viewpoint and lack a person-based approach.

The aim of the current study was to describe the person-based patterns of ambulatory care visits within Taiwan's National Health Insurance (NHI) program in 2002. Particularly, the quantitative analyses would concentrate on the frequency of switching physicians and healthcare facilities. The findings might provide evidence for discussion in health policymaking.

## Methods

### Data sources

A universal health insurance program has started in Taiwan since 1995 [4] and covered nearly all inhabitants (21,869,478 beneficiaries at the end of 2002, equivalent to a coverage rate of 97.1%) [5]. The NHI offers extensive hospitalization, ambulatory care and drugs with low co-payment rates. The ambulatory services of Western medicine, dentistry and traditional Chinese medicine belong to independent physicians' clinics and outpatient departments of hospitals. Although a specialty of family medicine does exist in Taiwan, the NHI beneficiaries are not required to register at a general practitioner as in British National Health Service system. The NHI does not establish a referral system of Western standard. The people have great freedom in choosing healthcare facilities and specialties. The outpatient departments, even at the academic medical centers of the tertiary care, are accessible at any time. The reimbursement within Taiwan's NHI is basically on a fee-for-service (fee for each item of services) basis under the global budgeting (similar to the German system). The hospitals and the NHI don't set the limit of visits within a day for each patient.

In 1999, the Bureau of National Health Insurance began to release all claims data in electronic form to the public under the project of National Health Insurance Research Database (NHIRD) [6]. Since then, dozens of extracted datasets has been available to researchers. The structure of the claims files had been described in details on the NHIRD web site and in other published works [7-9].

Every resident in Taiwan has a unique identification number officially and every healthcare facility in contract with the NHI has a unique identification number for claims purpose. The identification numbers of persons and healthcare facilities in the datasets have been encrypted to protect privacy. The encryption is consistent across all datasets so that the encrypted identification numbers remain unique, making longitudinal follow-ups feasible.

One special kind of datasets is the "cohort datasets" which include the claims data of 200,000 persons from 1996 to 2004 (status: November 2005). These 200,000 persons were randomly sampled from 23,753,407 persons who had ever been insured under the NHI from March 1, 1995 to December 31, 2000. The purpose of cohort datasets is to follow up a representative group of the population longitudinally. New claims data of the cohort would be released every year. Not every person of the cohort was insured throughout the whole period because of death and emigration. Besides, those who enrolled in the NHI at the first time after January 1, 2001 would not be included in the cohort.

According to the NHIRD, the randomization in sampling the cohort used the function (linear congruential random number generation) of Sun Work Shop C 5.0. The distributions of age, sex and utilization of the cohort were representative of the population. The data of residence and income were not available in the NHIRD datasets because the Bureau of National Health Insurance did not release such sensitive data.

Researchers who wish to access NHIRD datasets must sign a user agreement form indicating that they will obey related regulations and acknowledge the NHIRD in their publications. The study was approved by the Institutional Review Board of the Taipei Veterans General Hospital.

In the current study, we used only the ambulatory visit files of cohort datasets in 2002 (R{01..04}_CD2002.DAT). One record in the visit file might represent a consultation at the clinics or outpatient departments, a visit to emergency departments or preventive service, a prescription refill, or a use of ancillary services. There were a total of 2,572,065 visit records. One visit record contained up to three diagnostic codes in ICD-9-CM (International Classification of Diseases, Ninth Revision, Clinical Modification).

Besides, we used the registries for beneficiaries (R{01..04}_ID2002.DAT) and contracted healthcare facilities within the NHI (HOSB2002.DAT) to know the period of a person's eligibility for insurance and the accreditation category of healthcare facilities. We also used the registry for catastrophic illness patients (HV2002.DAT) to identify the patients with catastrophic illness. The updated registry for board-certified specialists (DOC2004.DAT) served to calculate the numbers of specialists per 100,000 people of the population.

### Study design

Among all ambulatory visit records in 2002, we calculated only those visits with physician consultations of western medicine, dentistry, and traditional Chinese medicine, including visits to emergency departments. We used the consultation fee to differentiate between a visit with physician consultation and visits merely for radiology, laboratory examinations, physical rehabilitation, or other ancillary services. Prescription refills, home care by nurses, and preventive services without physician consultation would also be excluded from analysis.

We at first analyzed the utilization patterns of ambulatory care visits at different specialties and at different types of healthcare facilities. The statistics were displayed both in numbers of visits and in numbers of patients. Within the NHI, 24 specialties and 22 subspecialties were recognized. In general, the subspecialties existed only at hospitals. We grouped the family physicians and those practitioners without any specialist title into general practice; otherwise, a subspecialty was deemed different from its main specialty. A healthcare facility with physician services was contracted with the NHI in one of 4 categories: academic medical center, metropolitan hospital, local community hospital, and physician clinic. Besides, we also calculated the distribution of principal diagnoses at all visits by ICD-9-CM chapter.

For person-based analyses, we calculated the numbers of visits, consulted specialties, physicians and healthcare facilities by each person during 2002. Their distributions of frequencies would be displayed. We also calculated the age-sex distribution in each group of patients by annual visit count.

The patients with catastrophic illness and the visits due to catastrophic illness would be separately identified and integrated into the analysis.

To investigate how frequently a patient might change physicians and healthcare facilities, we calculated the numbers of consultations in which the patient had visited other healthcare facilities on the same day, in the past 3 days, and in the past 7 days, respectively. We also calculated the numbers of patients with such help-seeking behaviors during the year. Furthermore, we calculated the numbers of consultations in which the patient had visited the same specialty at other healthcare facilities within the same time frames. Finally, we would try to quantify the "one-stop shopping" phenomenon, in which a patient might visit several specialties at the same hospital in a day.

### Data processing and statistical analysis

The open-source software Perl (version 5.8.6) [10] was used for computing. The regular statistics were displayed. The cohort originated from the people insured between 1995 and 2000. Because of death and emigration, not every person was still present in 2002. Our analysis was limited to the year 2002. In calculating the percentages of patients, the denominator was the number of persons who had been ever insured in 2002 according to the registry for beneficiaries.

## Results

### General information of the cohort

Among the 200,000-person cohort, only 182,474 persons were eligible for analysis: 90,447 women, 92,022 men and 5 persons of unknown sex. The mean age of the eligible persons was 34.7 (standard deviation [SD] 20.2) years. In 2002, the cohort had made 2,443,003 ambulatory care visits with physician consultation, and 14,136 (7.7%) persons did not have any visit.

### Number of visits by specialty, category of healthcare facility and principal diagnosis

General practice was the most popular specialty with 25.5% of all visits by 58.4% of the cohort in 2002. The top 5 specialties (general practice, otorhinolaryngology, Chinese medicine, internal medicine, and dentistry) together had a share of 61.6% of all visits (Table 1).

Although physician clinics had been visited by 87.4% of the cohort in 2002, the visits outside the hospitals made up only 69.6% of all visits (Table 2). A total of 105,007 (57.5%) persons had ever visited hospital-based outpatient or emergency departments in 2002. Emergency visits were relatively infrequent (1.8% of all visits), but many persons had ever used such services (16.2% of the cohort).

Diseases of the respiratory system were the most frequent diagnoses for visits. Almost one half of visits had either respiratory or digestive diseases as the principal diagnosis (Table 3).

Additionally, we had identified 5,419 (3.2%) patients with a certificate of catastrophic illness and 46,349 (1.9%) visits due to catastrophic illness among the cohort datasets. Patients with a certificate of catastrophic illness usually tended to have more visits. But, they accounted for only 13% (465 in 3,557) of patients with most frequent visits (*i.e. *> 52 visits annually).

### Frequency of visits, consulted specialties, physicians, and healthcare facilities per person of the cohort

On average, a person of the cohort had 13.4 (median: 10, 25 percentile: 4, 75 percentile: 19) physician consultations in 2002. Two fifths of the cohort had visited more than 12 times annually and made 76.3% of all visits together (Table 4). The age-sex distribution of the cohort by group of annual visit count was displayed in Table 5.

On the other hand, a person of the cohort had consulted 3.4 specialties, 5.2 physicians, and 3.9 healthcare facilities in 2002 (Table 6).

### Frequency of changing healthcare facilities within a short period

During the year, 80.8% (n = 147,372) of the cohort had visited two or more healthcare facilities. Furthermore, 17.3% of the cohort had ever visited different facilities on the same day (Table 7). In 2.1% of all visits, the consulted facilities should consider that the patient had visited other facility earlier on the same day. The percentage of second visits increased to 22.2% when the time frame became 7 days.

On the other hand, 49.7% (n = 90,678) of the cohort had visited physicians of the same specialty at different healthcare facilities in 2002. Likewise, 2.6% of the cohort had ever visited physicians of the same specialty at different facilities on the same day. When the time frame was 7 days, the percentage of the cohort with such a doctor-switching behavior increased to 23.5% and the share of second visits to 3.8%.

### "One-stop shopping"

During the year, 20.6% (n = 37,631) of the cohort had visited two or more specialties on the same day, and 6.2% (n = 150,392) of all visits belonged to such a type. About the "one-stop shopping" phenomenon, 7.6% (n = 13,819) of the cohort had visited two or more specialties at the same facility on the same day, and such visits make up 2.5% (n = 60,116) of all visits. Among these persons, 827 persons had ever visited 3 specialties at the same facility on the same day, and 54 persons had 4 specialties or more.

## Discussion

The strength of our current study was to take a person-based approach instead of the visit-based method seen in the National Ambulatory Medical Care Survey (NAMCS) in the USA [11]. The "patient-centered and population-based" [12] findings could reveal the percentage of people receiving any specific kind of medical care during a period of time, what was also known as "the ecology of medical care" [13,14]. In our study, we further expanded the traditional analyses to observe dynamic changes of seemingly static patient visits. Our study was purely descriptive. The question of doctor-shopping, open to judgment of value, could not be easily answered without further qualitative studies. But, we did have offered some clues.

First of all, the annual number of physician consultations per capita within the NHI in Taiwan was relatively high (13.4 visits). Among the member countries of the OECD (Organisation for Economic Co-operation and Development), only Japan and Hungary had exceeded Taiwan in this number (Japan: 16.0 in 1996; Hungary: 21.1 in 1999). By contrast, the American had only 5.8 visits in 1996 on average, the British 5.4 in 1998, the Canadian 6.4 in 1998, and the German 6.5 in 1996 [15]. On the other hand, the higher number in Taiwan might merely reflect higher availability and accessibility of medical care services in a densely populated island. The NHI in Taiwan succeeded not only in universal coverage of beneficiaries, but also in supply of extensive insurance benefits and contracted healthcare facilities. The low co-payment per visit (ranging from ca. 1.5 USD to 9 USD) did not form a barrier to access of medical care in general.

Secondly, the Taiwanese seemed to visit outpatient departments of hospitals frequently. In 2002, 69.6% of all ambulatory care visits were at physician clinics, 28.6% at outpatient departments, and 1.8% as emergency visits. By contrast, according to the National Health Care Survey in the USA [11], visits to physician offices accounted for 80.6% of all ambulatory care visits in 1999-200, while the outpatient departments of hospitals had only 8.6% of visits and the emergency departments as high as 10.8%. But the US data did not differentiate between community (secondary-care) and academic (tertiary-care) hospitals. The academic medical centers in Taiwan were possibly overloaded under the system of free access. The crowding at the hospitals could be only known in details from complete datasets rather than from person-based sampling datasets.

Thirdly, the average number of consulted physicians and healthcare facilities by an NHI beneficiary seemed to be high. Yet it might reflect either disproportional sub-specialization of physicians or lack of an established system of family physicians as "personal doctors" [16] in Taiwan.

Furthermore, the Taiwanese seemed to switch physicians and healthcare facilities frequently and quickly. A switch might be just a referral or visit for a second opinion. Most importantly, a physician in Taiwan should always keep in mind that in 3.8% of all visits the patient had visited other physician(s) of the same specialty at other facilities in the past week, no matter whether it was an explicit referral from medical professionals or an implicit self-referral by the patient.

The "one-stop shopping" phenomenon might be another feature of medical care in Taiwan. Because group practices with several specialties were uncommon in Taiwan, most visits to several specialties at the same facilities on the same day would likely occur at hospitals. That is, the share of such visits among all visits at hospitals would be nearly 8.1%. It might have resulted from either higher efficiency of hospital services or lack of integrated services. The practical meaning was that the time a patient stayed at the hospital would become longer.

Our study with insurance claims of the NHIRD had some limitations. First, the beneficiary's residence was not disclosed. The influence of location on ambulatory care utilization could not be studied. Second, the patient's complaints, symptoms, or other reasons for the visit were absent on NHI claims. Third, the claims also lacked fields of referral source and "visit disposition" that specified where the patient was referred from and what kind of follow-ups was planned. The conditions of switching physicians could thus not be verified.

The root of the revealed problems in our study might be the absence of an effective referral system in Taiwan, in other words, the absence of a coordinated system of health care delivery. The patients in Taiwan have unlimited rights of free choice of physicians and healthcare facilities. The primary care physicians are not used to writing referral letters and the hospital physicians are not used to issuing recommendation letters for other colleagues. A mechanism regulating data exchange between healthcare facilities does not yet exist. Not only the patient contacts become more complicated but also the people are unnecessarily exposed to contagious agents. The fundamental re-structuring of health care system will be a greater challenge to the policymakers in Taiwan.

## Conclusion

The people in Taiwan did visit the physicians and outpatient departments frequently. Many patients not only consulted several physicians of different specialties and at different healthcare facilities during the year, but also switched the physicians and facilities quickly. An effective referral system with efficient data exchange between facilities might be the solution.

## Abbreviations

NHI: National Health Insurance

NHIRD: National Health Insurance Research Database

ICD-9-CM: International Classification of Diseases, Ninth Revision, Clinical Modification

SD: standard deviation

NAMCS: National Ambulatory Medical Care Survey

OECD: Organisation for Economic Co-operation and Development

USD: United States dollar

NA: not available

## Competing interests

The author(s) declare that they have no competing interests.

## Authors' contributions

TJC conceived of the study, carried out the study, performed the statistical analysis and drafted the manuscript. LFC participated in the design of the study and helped to perform the statistical analysis as well as to interpret findings. SJH participated in the design and coordination of the study and helped to draft the manuscript. All authors read and approved the final manuscript.

## Pre-publication history

The pre-publication history for this paper can be accessed here:



## Figures and Tables

**Table 1:** Distribution of ambulatory care visits in 2002 by specialty (selected)

Specialty	No. of visits (%, n = 2,443,003)	No. of patients (%, n = 182,474)	No. of specialists per 100,000 population (in 2004)
General practice*	624,047 (25.5)	106,550 (58.4)	18.8**
Otorhinolaryngology	232,775 (9.5)	61,057 (33.5)	6.6
Chinese medicine	224,045 (9.2)	51,623 (28.3)	20.1
Internal medicine	218,191 (8.9)	54,144 (29.7)	28.6
Dentistry	204,773 (8.4)	68,959 (37.8)	43.3
Pediatrics	160,639 (6.6)	29,402 (16.1)	13.4
Ophthalmology	134,866 (5.5)	51,285 (28.1)	5.8
Gynecology	111,689 (4.6)	27,075 (14.8)	10.2
Dermatology	90,930 (3.7)	33,180 (18.2)	1.6
General surgery	72,394 (3.0)	26,899 (14.7)	19.8
Orthopedics	64,171 (2.6)	21,930 (12.0)	5.8
Cardiology	39,795 (1.6)	8,065 (4.4)	2.3
Gastroenterology	36,960 (1.5)	11,547 (6.3)	NA***
Neurology	35,052 (1.4)	8,526 (4.7)	2.8
Rehabilitation	31,981 (1.3)	7,953 (4.4)	2.6
Psychiatry	26,228 (1.1)	4,088 (2.2)	3.7
Urology	24,629 (1.0)	7,558 (4.1)	2.7
Infection	2,305 (0.1)	947 (0.5)	NA***
Others	107,533 (4.4)		

*General practice here included family medicine and the practice without any specialist title.

**Included only the board-certified specialists of family medicine.

***NA: not available.

**Table 2:** Distribution of ambulatory care visits in 2002 by contracted category of healthcare facilities

	No. of visits (%, n = 2,443,003)	No. of patients* (%, n = 182,474)
All visits		
Academic medical centers	224,838 (9.2)	39,613 (21.7)
Metropolitan hospitals	260,347 (10.7)	49,092 (26.9)
Local community hospitals	257,072 (10.5)	53,407 (29.3)
Physician clinics	1,700,746 (69.6)	159,499 (87.4)
Emergency visits		
Academic medical centers	12,050 (0.5)	8,768 (4.8)
Metropolitan hospitals	18,946 (0.8)	13,790 (7.6)
Local community hospitals	12,193 (0.5)	9,605 (5.3)
Physician clinics	271 (0.0)	230 (0.1)
Total	43,460 (1.8)	29,488 (16.2)
Outpatient visits		
Academic medical centers	212,788 (8.7)	36,901 (20.2)
Metropolitan hospitals	241,401 (9.9)	44,186 (24.2)
Local community hospitals	244,879 (10.0)	50,302 (27.6)
Total	699,068 (28.6)	98,894 (54.2)

*Because a patient might visit several healthcare facilities, the total of all percentages in this column exceeded 100%.

**Table 3:** Distribution of ambulatory care visits in 2002 by principal diagnosis

Diagnosis	Code range in ICD-9-CM*	No. of visits (%, n = 2,443,003)	No. of patients (%, n = 182,474)
Infectious and parasitic diseases	001–139	55,152 (2.3)	26,734 (14.7)
Neoplasms	140–239	34,307 (1.4)	8,598 (4.7)
Endocrine, nutritional, and metabolic diseases and immunity disorders	240–279	75,787 (3.1)	14,614 (8.0)
Diseases of the blood and blood-forming organs	280–289	4,520 (0.2)	1,917 (1.1)
Mental disorders	290–319	42,345 (1.7)	8,474 (4.6)
Diseases of the nervous system and sense organs	320–389	177,879 (7.3)	60,684 (33.3)
Diseases of the circulatory system	390–459	128,160 (5.2)	20,980 (11.5)
Diseases of the respiratory system	460–519	818,672 (33.5)	130,772 (71.7)
Diseases of the digestive system	520–579	370,761 (15.2)	102,540 (56.2)
Diseases of the genitourinary system	580–629	140,962 (5.8)	37,763 (20.7)
Complications of pregnancy, child birth, and the puerperium	636–076	6,890 (0.3)	2,953 (1.6)
Diseases of the skin and subcutaneous tissue	680–709	131,872 (5.4)	47,387 (26.0)
Diseases of the musculoskeletal system and connective tissue	710–739	171,817 (7.0)	45,227 (24.8)
Congenital abnormalities	740–759	2,799 (0.1)	1,063 (0.6)
Certain conditions originating in the perinatal period	760–779	94 (0.0)	64 (0.0)
Symptoms, signs and ill-defined conditions	780–799	129,573 (5.3)	48,930 (26.8)
Injury and poisoning	800–999	131,198 (5.4)	50,090 (27.5)
Supplementary classification of factors influencing health status and contact with health services	V01–V82	17,942 (0.7)	12,862 (7.0)
Supplementary classification of external causes of injury and poisoning	E800–E999	726 (0.0)	489 (0.3)
Unknown		1,547 (0.1)	655 (0.4)

*ICD-9-CM: International Classification of Diseases, Ninth Revision, Clinical Modification.

**Table 4:** Distribution of the beneficiary's visit frequency in 2002

Patient groups	Persons (n = 182,474)			Aggregate visits by each patient group (n = 2,443,003)		
		
No. of visits in 2002 per person	No.	%	Cum. %	No.	%	Cum. %
> 52	3,557	1.9	1.9	247,821	10.1	10.1
27 – 52	20,412	11.2	13.1	717,454	29.4	39.5
13 – 26	49,041	26.9	40.0	899,655	36.8	76.3
5 – 12	59,747	32.7	72.8	488,491	20.0	96.3
1 – 4	35,581	19.5	92.3	89,582	3.7	100.0
0	14,136	7.7	100.0			

**Table 5:** Age-sex distribution of the beneficiaries by visit frequency in 2002

Patient groups	No. of persons	Sex			Age (year)			
			
No. of visits in 2002 per person		Female	Male	Unknown	Mean	Median	25 percentile	75 percentile
> 52	3,557	1,880	1,677		54.4	63	43	73
27 – 52	20,412	11,610	8,802		42.8	46	19	64
13 – 26	49,041	27,801	21,240		35.5	35	17	51
5 – 12	59,747	30,145	29,601	1	31.7	30	18	44
1 – 4	35,581	14,364	21,216	1	32.2	30	20	43
0	14,136	4,647	9,486	3	35.8	35	24	46
Total	182,474	90,447	92,022	5	34.7	33	19	48

**Table 6:** Utilization of ambulatory care by number of consulted specialties, physicians, and healthcare facilities per person

	No. of persons	% (n = 182,474)	Cum. %
No. of consulted specialties per person in 2002			
> 12	212	0.1	0.1
7 – 12	15,648	8.6	8.7
6	13,197	7.2	15.9
5	21,104	11.6	27.5
4	29,041	15.9	43.4
3	34,414	18.9	62.3
2	31,944	17.5	79.8
1	22,778	12.5	92.3
0	14,136	7.7	100.0
No. of consulted physicians per person in 2002			
> 12	10,171	5.6	5.6
7 – 12	44,252	24.3	29.8
6	15,726	8.6	38.4
5	18,451	10.1	48.6
4	20,915	11.5	60.0
3	22,139	12.1	72.1
2	20,371	11.2	83.3
1	16,313	8.9	92.3
0	14,136	7.7	100.0
No. of visited healthcare facilities per person in 2002			
> 12	1,395	0.8	0.8
7 – 12	26,555	14.6	15.3
6	15,113	8.3	23.6
5	20,431	11.2	34.8
4	26,234	14.4	49.2
3	29,549	16.2	65.4
2	28,095	15.4	80.8
1	20,966	11.5	92.3
0	14,136	7.7	100.0

**Table 7:** Frequency of switching physicians and healthcare facilities in 2002

	No. of consultations (%, n = 2,443,003)	No. of patients (%, n = 182,474)
The patient has visited other healthcare facilities ...		
on the same day	52,359 (2.1)	31,646 (17.3)
in the past 3 days	324,377 (13.3)	87,851 (48.1)
in the past 7 days	542,256 (22.2)	105,570 (57.9)
The patient has visited the same specialty at other healthcare facilities ...		
on the same day	5,501 (0.2)	4,673 (2.6)
in the past 3 days	53,304 (2.2)	29,983 (16.4)
in the past 7 days	93,108 (3.8)	42,814 (23.5)
